# Improved Superconducting properties in the Mg^11^B_2_ low activation superconductor prepared by low-temperature sintering

**DOI:** 10.1038/srep25498

**Published:** 2016-05-05

**Authors:** Fang Cheng, Yongchang Liu, Zongqing Ma, M. Shahriar Al Hossain, M. Somer

**Affiliations:** 1 Tianjin Key Laboratory of Composite and Functional Materials, School of Materials Science & Engineering, Tianjin University, Tianjin 300072, People’s Republic of China; 2 Institute for Superconducting and Electronic Materials, AIIM, University of Wollongong, Squires Way, North Wollongong, NSW 2500, Australia; 3 Koc University, Chemistry Department, Rumelifeneri Yolu, TR-34450 Sariyer-Istanbul, Turkey

## Abstract

Mg^11^B_2_ has a great application prospect in the superconducting coils for fusion reactor as the “low activation superconductors”. The un-doped Mg^11^B_2_ and Cu-doped Mg^11^B_2_ bulks using ^11^B as a boron precursor were fabricated by low-temperature sintering in present work. It was found that the prepared Mg^11^B_2_ low activation superconductors exhibit better *J*_*c*_ performance than all of other Mg^11^B_2_ samples reported in previous studies. As for Cu doped Mg^11^B_2_, minor Cu addition can obviously improve the Mg^11^B_2_ grain crystallization and reduce the amount of MgO impurity. Hence, improved grain connectivity and higher *J*_*c*_ at low fields is obtained in Cu doped Mg^11^B_2_ samples. For un-doped samples, refined grains and more MgO impurity with proper size brought about more flux pinning centers, resulting in better *J*_c_ performance at high fields.

The observation of superconductivity in MgB_2_ at 39 K in 2001^1^ has attracted lots of attention. MgB_2_ shows excellent properties such as simple crystal structure, large coherence length, relatively high critical current density, and transparency of the grain boundaries and relatively low cost material. Using kinds of approaches, MgB_2_^2^,^3^,^4^,^5^,^6^,^7^,^8^,^9^ has been fabricated into bulks, single crystals, thin films, tapes and wires in various methods^10^,^11^. Taken together, these properties and preparation techniques support MgB_2_ to be a promising candidate for important large-scale and electronic device applications.

The radioactivity decay time of all kinds of superconducting wire^12^ who had broad application prospect in the international thermonuclear experimental reactor (ITER) Tokamak superconducting coil showed that Nb-based superconductor wires who have been widely used nowadays had a very long induction of radioactive decay time, so Nb-based superconductor wires needed much longer time to cool down. The longer cooling time brought negative impact on the environment and treating processes. Furthermore, despite their relatively high *J*_*c*_*s*^13^,^14^,^15^, Nb-based superconductors suffer high operation costs due to the usage of liquid helium as coolant. In contrast, MgB_2_ radioactivity decay time is very short and has higher running temperature, so it has a great application prospect in the fusion reactor as the “low activation superconductors”. In general, the natural elementary boron is composed of 20% ^10^B and 80% ^11^B. In the irradiation environment, the reaction ^10^B + n → ^7^Li + He (gas) will occur due to the large thermal neutron capture cross section of ^10^B. Hence, as part of superconductor, there is no guarantee for the long-term stability of the MgB_2_ superconducting magnet. Isotope research shows that ^11^B is much more stable than ^10^B in neutron irradiation environment due to the smaller neutron capture cross section^16^ which can ensure the operation stability of MgB_2_ superconductor magnet. Previous studies have been focused on the theoretical calculation of isotope boron effect on the MgB_2_^17^,^18^, but little on the experiment research. Only a few previous experimental studies put many emphases on the performance of the Mg^11^B_2_ superconducting wires^19^,^20^, which performed obviously different from the traditional MgB_2_ wire with natural B powder, and the critical current density exhibits much lower than the traditional MgB_2_ with natural B powder^20^.

On the other hand, one can know that the traditional MgB_2_ samples using natural boron powder which fabricated at low temperature generally had higher critical current density at high fields than those prepared at high temperature due to inferior crystallinity^21^,^22^,^23^. However, the reaction at low temperature generally took a very long time to form the complete MgB_2_ because of the low diffusion rate of atom in the solid-state below the melting point of Mg^24^. Y. Hishinuma *et al.* also reported that the successful synthesis of Mg^11^B_2_ samples with higher *J*_*c*_ performance needs to take as long as 200 h by heating at 500 °C^20^. From this view, it makes sense to rapidly fabricate Mg^11^B_2_ samples with improved *J*_*c*_ by heating at low temperature.

In previous studies, the influence of Cu addition on the sintering process and superconducting properties of MgB_2_ samples with natural boron powder were systemically studied^25^,^26^ and it was found that minor Cu addition was proved to benefit to the formation of MgB_2_ phase at low temperature, and thus MgB_2_ bulks with excellent *J*_*c*_ performance can be rapidly fabricated at low temperature by Cu activated sintering^26^,^27^. Inspired by this, herein the influence of Cu addition on the Mg^11^B_2_ bulks with isotope ^11^B powder will be studied and Cu addition is expected to benefit to its low temperature sintering process as well as superconducting properties. Actually, the research about the influence mechanism of Cu addition on the Mg^11^B_2_ bulks is seldom carried out till now.

Based on these backgrounds, in present study, we focused on the synthesis of Mg^11^B_2_ with higher superconducting properties by heating at low temperature for short holding time. In this study pure ^11^B was used as the raw material instead of natural B, with the purpose of solving the tokamak plasma problem in controllable thermonuclear fusion. Besides the un-doped samples, we also investigated the phase formation and superconducting properties in the Cu-doped Mg^11^B_2_ samples. Combined with the measurement of the *T*_*c*_ and critical current density, the effects of the element ^11^B and Cu addition on the superconducting properties of MgB_2_ are concluded.

## Experimental

The samples of un-doped Mg^11^B_2_ and Mg^11^B_2_ + 5wt.% Cu were prepared by a solid-state sintering at low temperature. ^11^B powders (Amorphous, about 5 μm in size, from Pavezyum Kimya, Turkey), Mg powders (99.5% purity, 100 μm in size), and Cu powders (99.7% purity, 3 μm in size) were separately mixed in the ratio of Mg^11^B_2_ and Mg^11^B_2_ + 5wt.% Cu. Then, the mixed powders were pressed into cylindrical pellets (*Ф*5 × 1.5 mm). All the un-doped and Cu-doped pellets were sintered in differential thermal analysis (Mettler Toledo TGA/DSC1/) respectively at 600 °C for 5 h, 10 h and 15 h under flowing high-purity Ar gas with a heating rate of 10 °C min^−1^ and then cooled down to room temperature with a cooling rate of 40 °C min^−1^. The phase composition of the sintered samples was examined by X-ray diffractometer (XRD, Rigaku D/max 2500) using Cu Kα radiation, and the morphology of the samples was characterized by scanning electron microscope (SEM, S-4800, Hitachi). The superconducting properties were measured by superconducting quantum interference device (SQUID VSM, Quantum Design), after the samples were cut into a slab of size about 4 × 2 × 1 mm^3^. The corresponding *J*c values were calculated from the width of magnetization hysteresis loops based on the Bean model *Jc* = *20*Δ*M*/[*a*/(*1* − *a*/*3b*)]^28^, where Δ*M* is the volume magnetization, and *a* and *b* are the sample dimensions.

## Results and Discussion

**Figure 1:**
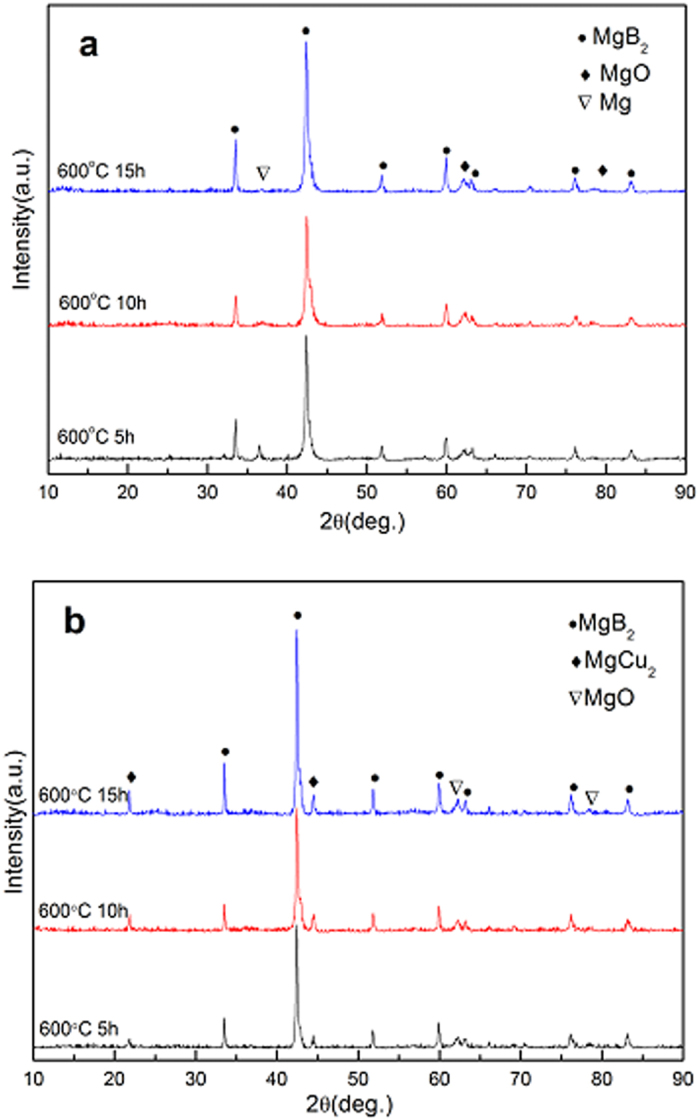
The XRD patterns of samples sintered at 600 °C for different heating time (**a**) un-doped Mg^11^B_2_ and (**b**) Cu-doped Mg^11^B_2_ samples.

Figure 1 presents the XRD patterns of the un-doped and Cu-doped Mg^11^B_2_ samples sintered at 600 °C for 5 h, 10 h and 15 h respectively. In the un-doped samples (see Fig. 1a), one can see that MgB_2_ is the main phase which is different from the traditional MgB_2_ sample with natural boron powder sintered at the same condition. In previous studies^25^,^27^, it was reported that no obvious MgB_2_ peaks can be observed in the un-doped MgB_2_ samples sintered below 650 °C only if some additional methods are employed, such as chemical doping^29^,^30^. Besides, the peaks of residual Mg phase in the un-doped samples decrease as the holding time prolongs, indicating that longer sintering time could facilitate the reaction between Mg and ^11^B forming Mg^11^B_2_ at low temperature. On the other hand, the residual Mg peaks cannot be recognized in all the Cu-doped samples. This implies that Cu addition can significantly promote the formation of Mg^11^B_2_ phase, just as the case of MgB_2_ samples with natural boron powder^25^,^29^. Local Mg-Cu liquid resulting from the eutectic reaction between Cu and Mg at about 485 °C can enhance Mg diffusion and thus accelerate the formation of MgB_2_ phase^25^,^26^. Furthermore, comparing with the un-doped ones, the Cu-doped samples contain less MgO impurity. The decrease of MgO impurity owes to the existence of local Mg-Cu liquid which could wrap the neighboring Mg particles and thus protect them from the oxidation^31^.

**Figure 2:**
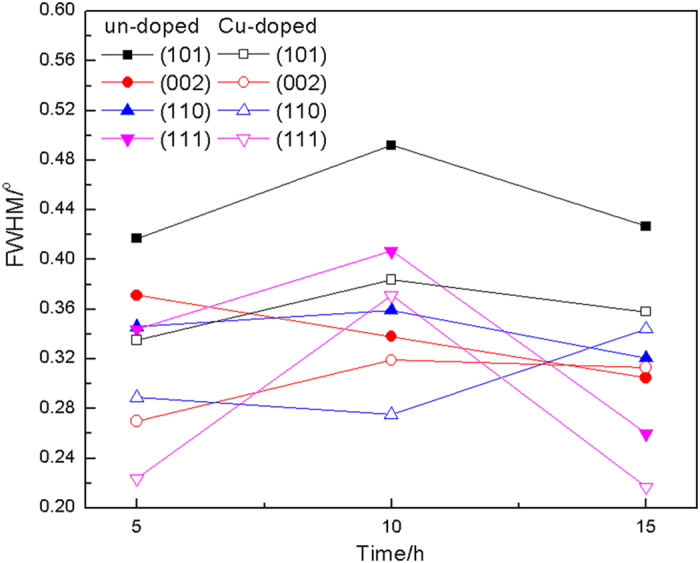
FWHM of the un-doped and Cu-doped samples prepared at 600 °C for 5 h, 10 h and 15 h. The corresponding planes of MgB_2_ are (101), (002), (110) and (111), respectively.

Based on the X-ray diffraction patterns of samples, the full width at half maximum (FWHM) was obtained and shown in Fig. 2. One can see that the FWHM of MgB_2_ peaks in the Cu-doped samples are smaller than that of un-doped ones. This result demonstrates that the size of MgB_2_ grains in the Cu-doped samples is larger than un-doped ones. In previous studies^10^,^11^,^31^, it was reported that the minor Cu addition can promote the growth of grain and improve the crystallization degree.

The sintering density of samples is present in Table 1. The density of all sintered samples is similar and about half of the theoretical value (2.62 g/cm^3^), but higher than the other ones in previous studies using the similar preparation technique^32^. Generally, the chemical reaction between Mg and B forming MgB_2_ results in about 30% volume reduction and porosity is known to be difficult to avoid during the sintering process^33^. This is the main reason why the *in-situ* sintering density of Mg^11^B_2_ bulks is generally lower than their theoretical value. In addition, the inevitable evaporation and oxidation of Mg during sintering process also have a negative impact on the sintering density. Interestingly, the sintering density of Cu-doped Mg^11^B_2_ samples was a little higher than that of un-doped samples. It could be explained that more sintering densification can occur in Cu doped samples due to the existence of local liquid sintering environment resulting from Mg-Cu liquid at low temperature. Besides, since the MgCu_2_ impurity as a result of Cu addition possesses relatively higher density than MgB_2_, finally it can contribute to the higher sintering density of Cu doped samples.

**Figure 3:**
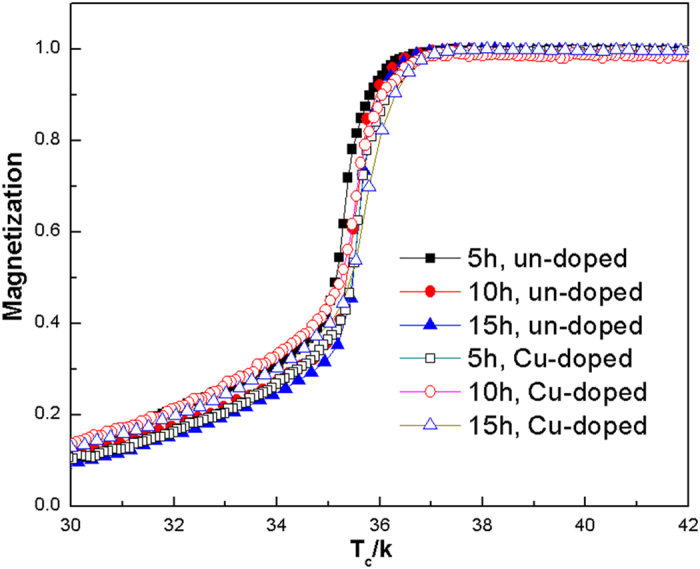
Temperature dependences of ZFC magnetization for the sintered samples.

The temperature dependence of magnetization curves for the un-doped and Cu-doped Mg^11^B_2_ samples were shown in Fig. 3, and the corresponding results were listed in Table 1. The transition temperature (*T*_*c*_) of Mg^11^B_2_ samples is about 36 K, which is generally lower than the normal MgB_2_ due to the isotope effect of ^11^B^17^,^18^. But the critical temperature of our Mg^11^B_2_ bulks is comparable to other Mg^11^B_2_ samples^20^, indicating that isotope effect is the key factor lowering *T*_*c*_, rather than other processing conditions. It is worth noting that the critical temperature of all our samples is above 35 K which still meets the actual needs of practical applications. Besides, the transition width (Δ*T*), the interval between *T*_c, onset_ and *T*_c, end_, becomes broad as the sintering time prolongs. As all know, the high critical temperature and sharp transition width indicate that the samples are of high crystallinity and homogeneity. It is suggested that the increased sintering time can facilitate the reaction between Mg and B completely. With extend of holding time, indefinite quantity of impurities can be introduced, which may reduce the purity and homogeneity of samples.

**Figure 4:**
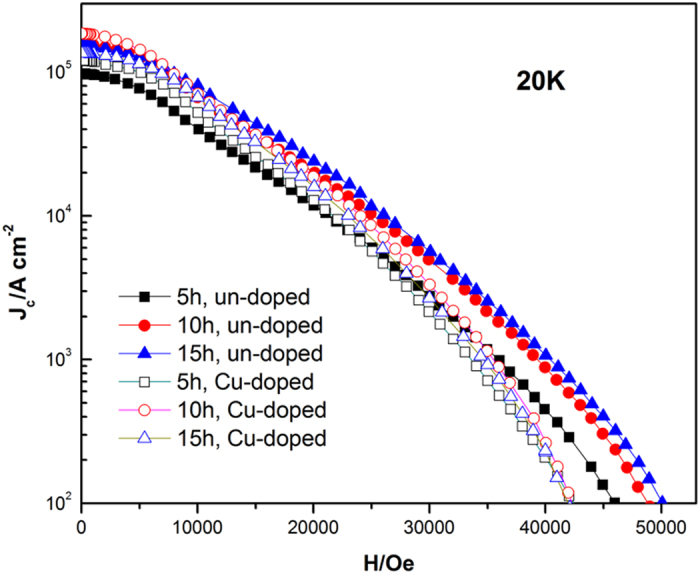
The measured *J*_*c*_*-H* characteristics of the sintered samples at 20 K.

The critical current density (*J*_*c*_) and irreversibility field (*H*_irr_) of samples are illustrated in Fig. 4 and Table 1. The samples with Cu addition showed relatively lower *J*_c_ than the un-doped samples at high field, whereas higher *J*_c_ at low-field. Generally speaking, critical current density in MgB_2_ is decided by both grain connectivity and flux pinning^34^,^35^. As discussed above, minor Cu addition can obviously improve the Mg^11^B_2_ grain crystallization and reduce the amount of MgO impurity. Hence, improved grain connectivity and higher *J*_*c*_ at low fields is obtained in Cu doped Mg^11^B_2_ samples. On the other hand, minor Cu addition can also lead to the grain growth, implying that grain boundary pinning becomes weakened in Cu doped sample compared to undoped one. Moreover, more MgO impurity is formed in un-doped samples and some of them with proper size can also serve flux pinning centers. Consequently, better *J*_c_ performance at high fields is expected in undoped samples. From Table 1, one can see that the value of *J*_*c*_ and *H*_*irr*_ is increasing with the increasing sintering holding time. Besides, compared to previous studies^20^, the *J*_*c*_ value is higher while the sintering time is shorter due to the state of ^11^B which has smaller size and more activity. These advantages can accelerate the reaction rate and reduce the the raw materials exposure to oxygen, so it’s beneficial to refine the grains and get much purer samples. On the whole, the un-doped sample for 15 h possesses the optimal performance in superconductivity. In order to manifest the cause, we have accomplished the following measurement.

**Figure 5:**
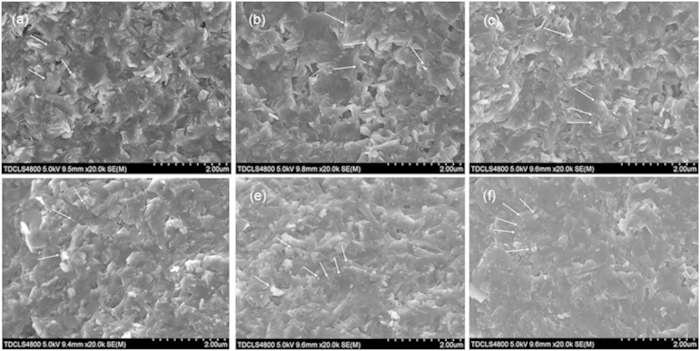
SEM images for the un-doped sintered for (**a**) 5 h, (**b**) 10 h, (**c**) 15 h, and the Cu-doped Mg^11^B_2_ samples sintered at (**d**) 5 h, (**e**) 10 h, (**f**) 15 h.

The SEM images of un-doped and Cu-doped Mg^11^B_2_ samples are illustrated in Fig. 5. From the SEM images, the size of MgB_2_ grains is larger in the Cu doped samples (about 600 ~ 800 nm) than that of un-doped samples (about 500 ~ 600 nm) and their individual grains with clear shape are easier to distinguish. In addition, the degree of crystallinity of Cu-doped samples is better than un-doped samples. These results indicate that slight Cu addition has a positive effect on the growth of grain and the improvement of crystallinity, which can enhance grain connectivity. And therefore, the *J*_*c*_ value of Cu-doped samples was improved at low field. The oxide particles, as the main impurities, are marked by the white arrows in these SEM images so that they can be recognized clearly. One can see that mass of oxide particles can be observed in sintered samples, especially in undoped samples. As holding time prolongs, the amount of these oxide particles gradually increase, consistent with the XRD results. It is explained that as the holding time increases, More MgO impurity can form for the reason that Mg further reacts with the trace oxygen in flowing Ar protective gas. Due to the small size of our Mg^11^B_2_ bulks (*Ф*5 × 1.5 mm), even the trace oxygen in the flowing Ar protective gas can result in relatively large amount of MgO impurity. Observing carefully, one can also see that the size of MgO particles became smaller and their distribution became more homogeneous with the extended holding time. It is worth noting that these small MgO particles may serve as the flux pinning centers as their size is comparable to the coherent length of pure MgB_2_.

**Figure 6:**
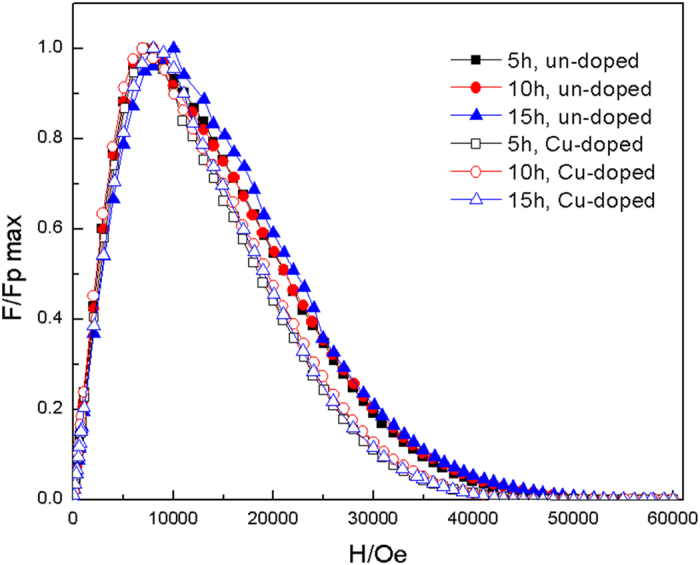
Normalized flux pinning force density as a function of applied magnetic field for the Mg^11^B_2_ samples.

Figure 6 shows the normalized pinning force *F*/*F*_*p max*_ of Mg^11^B_2_ samples in magnetic field. One can see that the pinning peaks of un-doped samples moved to high field with the increasing holding time. This result suggests that the pinning force in the un-doped samples increases as the sintering time prolongs. Combined with the results above (as shown in the Fig. 5a–c), it is explained that there are more MgO impurity with the size comparable to the coherence length of MgB_2_ in the un-doped samples, which is helpful to get and establish more pinning centers^36^.

## Conclusions

In present work, the Mg^11^B_2_ samples were synthesized by low-temperature sintering. The value of *J*_*c*_of un-doped samples at high fields is higher than Cu-doped ones, whereas Cu-doped samples exhibit better *J*_*c*_ performance at low fields. Minor Cu addition can significantly promote the growth of Mg^11^B_2_ grains as well as decrease the amount of MgO impurity. Consequently, higher sintering density and improved grain connectivity are obtained in Cu doped Mg^11^B_2_ samples, which contribute to the better *J*_*c*_ performance at low fields. On the other hand, larger grain size and less MgO impurity can also decrease the flux pinning centers, and thus result in the lower *J*_*c*_ at high fields. For un-doped samples, unconspicuous growth of grain size and more volume fraction of MgO brought about the increase of flux pinning centers, hence, the un-doped samples show higher *J*_c_ performance at high field.

## Additional Information

**How to cite this article**: Cheng, F. *et al.* Improved Superconducting properties in the Mg^11^B_2_ low activation superconductor prepared by low-temperature sintering. *Sci. Rep.* **6**, 25498; doi: 10.1038/srep25498 (2016).

## Figures and Tables

**Table 1:** The superconducting properties of the samples.

Sample		*ρ*(*g*/*cm*^3^)	*Tc*(*K*)	*∆T*(*K*)	*H*_*irr*_ (*T*)	*J*_c_ at 3T, 20 K (×10^3^ A·cm^−2^)
Un-doped	5 h	1.30	35.75	0.82	4.61	2.815
	10 h	1.29	35.88	0.77	4.88	4.881
	15 h	1.31	36.17	1.03	5.01	5.782
Cu-doped	5 h	1.39	35.91	0.69	4.24	2.106
	10 h	1.31	36.01	1.05	4.23	3.395
	15 h	1.34	36.28	1.41	4.19	2.748
